# Separation of Heart and Lung-related Signals in Electrical Impedance Tomography Using Empirical Mode Decomposition

**DOI:** 10.2174/1573405618666220513130834

**Published:** 2022

**Authors:** Kuo-Sheng Cheng, Po-Lan Su, Yen-Fen Ko

**Affiliations:** 1 Department of Biomedical Engineering, National Cheng Kung University, Tainan 701, Taiwan;; 2 Department of Internal Medicine, National Cheng Kung University Hospital, College of Medicine, National Cheng Kung University, Tainan 701, Taiwan;; 3 College of Biomedical Engineering, China Medical University, Taichung 404, Taiwan

**Keywords:** Empirical mode decomposition, principal component analysis, lung EIT, V/Q, bedside monitoring, EIDORS

## Abstract

***Background*:** Electrical impedance tomography (EIT) can be used for continuous monitoring of pulmonary ventilation. However, no proper method has been developed for the separation of pulmonary ventilation and perfusion signals and the measurement of the associated ventilation/perfusion (V/Q) ratio. Previously, various methods have been used to extract these components; however, these have not been able to effectively separate and validate cardiac- and pulmonary-related images.

***Aims*:** This study aims at validating and developing a novel method to separate cardiac- and pulmonary-related components based on the EIT simulation field of view and to simultaneously reconstruct the individual images instantly.

***Methods*:** Our approach combines the advantages of the principal component analysis (PCA) and processes that originally measure EIT data instead of handling a series of EIT images, thus introducing the empirical mode decomposition (EMD). The PCA template functions for cardiac-related imaging and intrinsic mode functions (IMFs) of EMD for lung-related imaging are then adapted to input signals.

***Results*:** The proposed method enables the separation of cardiac- and lung-related components by adjusting the proportion of the key components related to lung imaging, which are the fourth component (PC4) and the first component (IMF1) in PCA- and EMD-based methods, respectively. The preliminary results on the application of the method to real human EIT data revealed the consistently better performance and optimal computation compared with previous methods.

***Conclusion*:** This study proposes a novel method for applying EIT to evaluate the best time of V/Q matching on the cardiovascular and respiratory systems; this aspect can be investigated in future research.

## 1 INTRODUCTION

Electrical Impedance Tomography (EIT) can provide real-time and continuous monitoring of pulmonary ventilation. However, no proper method has been developed for the simultaneous separation of pulmonary ventilation and perfusion signals or for the measurement of the associated ventilation/perfusion (V/Q) ratio. This study aims at validating and developing a novel method to separate cardiac- and pulmonary-related components based on the EIT simulation field of view and to simultaneously reconstruct the individual images instantly. Additionally, this study proposes a novel method for applying EIT to evaluate the best time of V/Q matching on the cardiovascular and respiratory systems in clinical practice.

There is no direct evidence to evaluate the lung-achieved V/Q matching. It is difficult to monitor ventilation and perfusion at the same time in clinical practice. While Positron Emission Tomography and Computed Tomography can be applied to lung imaging, they cannot be used for continuous monitoring at the bedside.

The V/Q ratio determines the adequacy of gas exchange in the lung. In respiratory therapy, appropriate pressure is applied to the patient in order to open the airway for breathing and hence reduce V/Q mismatching. However, the V/Q ratio is not readily available to the clinician.

Optimal Positive End-Expiratory Pressure (PEEP) strategy can improve the cure rate and reduce mortality. Deciding when to apply PEEP is easy, but it is difficult to determine how much PEEP to use.

The setting of pressure levels is based on the physician’s insights to determine the optimal PEEP mode and on physiological parameters, such as blood oxygen, PaO2, SpO2, and blood pressure that are used to evaluate the ability of pulmonary gas exchange.

Many strategies have been proposed for PEEP titration to arrive at the optimal PEEP setting based on an individual’s need. However, so far, none has been conclusive.

Recently, EIT has been used as a bedside monitoring tool during ventilation therapy to observe the distribution of lung impedance change. Many studies have utilized EIT to guide PEEP and to optimize the strategies for individual needs. Most past studies of lung EIT have focused on ventilation-induced impedance change, ignoring perfusion-induced impedance.

The electrical impedance image contains information on pulmonary blood perfusion. It is a challenge to separate the weaker perfusion-related impedance changes than it is for the ventilation-induced impedance changes. Therefore, McArdle *et al.* proposed the first approach for the separation of ventilation- and perfusion-related impedance changes [1].

So far, the following five methods have been proposed to extract the perfusion signal or separate these two signal methods: breath-holding, injecting a contrast agent, ECG-gating, Fourier spectrum-based, and PCA-based methods.

### 1.1 Breath-Holding and Injecting a Contrast Agent

The simple and straightforward way to avoid interference between each other is breath-holding during EIT measurements. Otherwise, using an invasive way to enhance the cardiac-induced impedance changes is by injecting saline solution as a contrast agent [2, 3]. However, these methods are not feasible in the long term and for real-time bedside monitoring, and the ventilation-induced conductivity changes are suppressed.

### 1.2 ECG-Gated EIT Method

ECG-gated EIT method was the first approach proposed by McArdle *et al.* 1988 [1] to separate the cardiac and respiratory changes in EIT applications. This technique uses an electrocardiogram to synchronously measure ECG signals during EIT measurements and average several cardiac cycles. Finally, it amplifies the perfusion-related signal of the mixed EIT-signal. In other words, a long period of data from the cardiac cycles is required as a trigger [4]. Therefore, the method is not suitable for real-time monitoring and sudden changes and irregularities or abnormalities cannot be detected [5]. Furthermore, considering this method of extraction, the component of ventilation-induced conductivity changes is lost [6].

### 1.3 Fourier-Spectrum-Based Method

While attempting to filter either of the reconstructed image sequences, one must take advantage of the differences between the frequency characteristics of the respiratory and heart rates [7, 8]. There are well-separated results for the frequencies of the respiratory and heart rates if there is no overlap between the rates [9]. However, the results are often affected by the frequency overlapping of the ventilation harmonics and the cardiac signals. Hence, the extracted results still interfere with each other, and they are present in the image even after filtering.

### 1.4 PCA-Based Method

PCA is based on the multivariate statistical method, which projects the EIT data stream to an orthonormal basis. The advantage of this method is that there is no dependence on the ECG or any further instrumentation as a prior knowledge. So far, most investigations use the PCA technique to decompose the EIT image sequences into their parts, namely perfusion and ventilation. In other words, the technique requires a period of EIT data frames to calculate the coefficient matrix and template function.

Methods proposed in the past have tried to separate both parts in the EIT reconstructed image sequences. However, in doing so, the separated or extracted results lose some ventilation or perfusion information. This occurs because the reconstructed image uses electrode signals that mix EIT signals to solve the ill-posed inverse problem. Moreover, because of lung expansion, the cardiac-related imaging suffers from interferences at the cardiac signal level, which is much below the ventilatory signal level.

Furthermore, present methods use post-processing operation and are impractical in real-time. Studies have been published on separating ventilation- and perfusion-related information from EIT data streams in real EIT applications. However, they have produced incomplete results on spatial or temporal resolution; thus, the determination of a more reliable method has been inconclusive.

However, only a few studies have reported simulations and phantom experiments to address the issue of the inverse problem. Furthermore, separating the desired signal from the raw voltage data (electrode signals) between electrodes before reconstructing an image has not yet been studied.

In this study, we performed the Finite Element Method (FEM) model and EIT simulation experiments. We proposed a novel method that combines the use of PCA and EMD for extracting individual ventilation and perfusion-related components before reconstructing an EIT image and subsequently solving the inverse problem.

As shown in Fig. (**1**), there is a brief comparison between the general reconstruction and extraction procedures in the form of a flowchart.

The comparison is based on different methods (ECG-gated, Fourier-spectrum, PCA) used as post-processing techniques and the proposed method, based on the novel modified separation method, as a pre-processing technique. Additionally, we apply the proposed modified separation method to test the real EIT data.

## 2 MATERIALS AND METHODS

To verify the proposed separation algorithm, three-dimensional EIT simulations and image reconstructions were designed to test the PCA- and EMD-based methods. The FE model designed for phantom experiments consists of three spheres, representing the heart and lungs that are shown as the thorax in Fig. (**2**). The FE model was a 3D cylinder with 16 electrodes located at the boundary. The radius of the left and right spheres ranged from one to two times the radius of the middle sphere.

The EIT simulations used an adjacent pair for current injection and voltage measurements. For each current injection pair, 13 voltages were measured from the adjacent electrodes. After the measurement at all the 16 pairs, 208 data points were obtained for further image reconstruction.

To characterize every component, all the 208 data points were first decomposed and analyzed using PCA-based and EMD-based methods before the reconstruction process. Only the heart- and lung-related components were selected to remodel the measured data to reconstruct the lung and heart images separately. Sophisticated phantom models were generated using the FEM modeling software Netgen 5.3. Further, the forward as well as the inverse problems were solved using the EIDORS v3.8 to reconstruct and display the EIT images.

### 2.1 FEM Phantom Experiments

Fig. (**1**) shows the designed FEM phantom, which includes 16 electrodes and three spheres that contain a lung region of conductivity 0.5 as well as a heart region of conductivity 2. The radius of the heart is 0.3 while the radius of the lung ranges from 0.3 to 0.6 (unit: arbitrary). The proposed method combines two major techniques, which will be briefly introduced.

### 2.2 Principal Component Analysis

PCA is an unsupervised multivariate statistical method that transforms data into a direction of maximal variance and eliminates redundancy. The data are decomposed into multi-dimensions, which include eigenvectors and eigenvalues: the former values are measured for denoising, and the latter are listed in descending order to show the importance and variance as such.

In previous approaches, the PCA was commonly used to identify template functions with a sequence of image data. It was utilized to extract the cardiac or ventilatory-related impedance changes. In the proposed approach, the PCA is chosen to decompose the measured data, wherein the input signal contains 208 voltage data points for imaging.

Before transforming the data to obtain the template functions, the dimension of the input signal must be reshaped from 208 × 1 to 13 × 16 as the input signal matrix X.

The columns in the X-matrix correspond to each current injection pair, and the rows correspond to measurements of 13 voltage pairs for each current injection pair. The PCA resulted in a principal component covariance matrix C in which the diagonal elements are sorted by descending the variance and covariance between off-diagonal elements. This was approximately equal to zero. Thereafter, matrix C was used to project the data X into the eigenvectors. This is a transformation of X in the principal component space and represents the score of each principal component, termed as matrix PC:


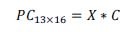


Finally, we reconstruct new data X-using the cardiac- or ventilatory- related eigenvectors with i = cardiac- or ventilatory-related components respectively, as expressed below:


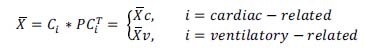


### 2.3 Empirical Mode Decomposition

EMD is a useful analysis method that breaks down the non-linear or non-stationary signal into characteristic components called intrinsic mode functions or IMFs. Each IMF represents a different part of the signal that carries the same frequency information at different time durations. In a recent study, EMD was utilized as a new method for analyzing physiological signals, and it has shown good results for extracting features [10-14].

The advantage of the EMD method is that it provides an appropriate approach to solve the non-linear ill-posed inverse problem for EIT reconstruction. In the present approach, the EMD is chosen to decompose the measured data that has 208 voltage data points.

EMD is used to decompose the measured EIT data into a finite number of IMFs. This can be expressed as follows:


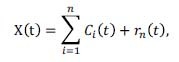


Where X (t) is one set of the serial EIT measured data, C_i (t) is ith IMF, and r_n (t) is the residue.

Finally, we reconstructed new data (X (t)) ®, using the cardiac- or ventilatory- related IMFs with i = cardiac- or ventilatory-related combinations respectively as demonstrated below:


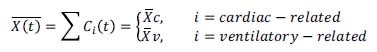


Fig. (**3**) illustrates the method used to generate FEM phantom and obtain the corresponding voltages. The PCA- or EMD-based separating approach is applied before solving the inverse problem.

For each simulated EIT, the radius of the lung region was adjusted to generate a different size of the lung and the forward problem was solved to obtain the corresponding 208 voltage data points. The PCA- and EMD-based methods were then used to decompose these data points into several components and 208 new voltage data points with cardiac- and ventilatory-related combinations were recomposed. These voltage data points were used to solve the inverse problem and reconstruct the EIT image for heart and lung imaging, respectively. Finally, the reconstructed heart and lung images were displayed separately.

### 2.4 Error Estimation

We estimate the following parameters. A group of experts in EIDORS developed a set of figures of merit to characterize the performance of an algorithm for GREIT test parameters [15].

Various error functions have been proposed to estimate the performance figures of merit. These functions include Amplitude response (AR), Position error (PE), Resolution (RES), Shape deformation (SD), and Ringing (RNG). Among these consensus definitions, PE, SD, RNG were chosen to calculate and estimate the proposed PCA-based and EMD-based separation algorithm. These parameters were used in this study for the following purposes:

PE estimates the error of position between the center of the simulation target and the center of the reconstructed image. In this study, PE was used to evaluate the performance of separating heart and lung images to achieve the desired location. Therefore, the minimum value of PE is 0, representing the reconstructed results at an expected location.

SD estimates (calculates) the match blob to equal the circular area. In this case, SD was used to evaluate the variance of separating heart and lung imaging from the expected shape. Therefore, the desired SD is 0, demonstrating that the reconstructed results fit in the expected position and shape.

RNG calculates the amplitude ratio of the inverted reconstructed image area. In this study, in a high SD case, the high RNG represented the reconstructed result, which was an undesired image because of the inverted main reconstructed image. When the reconstructed image has low or no SD, the RNG reflects the transition zone's response, which converges toward the desired image. A higher RNG indicates a better performance of the separation that responds rapidly, and the reconstructed image is more similar to the simulated FEM model.

This shows the corresponding outcome from the observed horizontal center cut (profile) of the reconstructed images, and it is compared with the above error parameters.

The differences in error parameters could be observed from the reconstructed images and the horizontal center cut.

Moreover, the results of reconstructed images, horizontal center-cut, and the error parameters should be consistent with one another.

This study was partially funded by research grant from the Ministry of Science and Technology of Taiwan (http://www.most.gov.tw) (108-2221-E-006-176).

## 3 RESULTS

### 3.1 Overview

A total of 208 measured data points from the phantom were decomposed using the PCA and EMD methods. One of the components was removed from the sequence and the remaining components were recomposed to reconstruct an EIT image.

### 3.2 Observations

The heart and lung impedance-related change from the horizontal central cut of reconstructed images was observed to obtain the characteristics of each component. Based on the observations, the components related to the heart or the lungs were selected to recompose and reconstruct the heart and lung EIT images, respectively. A human dataset was used to demonstrate the performance of the separation algorithm. The human dataset was captured using the EIT scanner designed by GhislainSavoie and Robert Guardo; the scanner is a 16-electrode EIT system with an adjacent drive.

Fig. (**4**) shows the state of the gradual expansion of the lungs during simulated breathing. The spheres of the FEM have a fixed heart radius of 0.3, while the lung radius ranges from 0.3 to 0.6 (unit: arbitrary). As shown in Fig. (**4a**), the heart and the lung regions can be seen clearly from the imaging when the radius of the lung is equal to that of the heart. Fig. (**4b**) shows that the heart region in the imaging was suppressed when the lungs were expanding. Figs. (**4c** and **d**) show the horizontal central cut of the imaging. The blue peak shifted downward, indicating that lung expansion affected heart imaging.

### 3.3 Heart Separation

#### 3.3.1 PCA-Based Reconstruction

A total of 208 measured data points were analyzed by the PCA-based method and decomposed into 16 components. According to the accumulated eigenvalue, the first eight components contained more than 95% of the information. The modified measured data were recomposed using the first to eighth (or more) components and used to reconstruct the image.

The resulting horizontal center cut of the image is shown in Fig. (**5**). As shown in Fig. (**5**), the blue line illustrates the reconstructed image with complete distortion, and the green line is similar to the original reconstructed image.

The result indicates that the first to twelfth components must be included in the recomposition process. Otherwise, the reconstructed EIT imaging will be distorted.

After removing one of the selected 12 components, the remainder is recomposed to reconstruct the images to identify the components' characteristics.

In Fig. (**6**), the horizontal center cut of the images shows that the reconstructed image, except the first, second, fifth and sixth components, will be completely distorted, and the heart and lung regions are reversed, as shown in Figs. (**6a, b, e** and **f**). This phenomenon indicates that the first, second, fifth, and sixth components are highly correlated with reconstruction. Therefore, these components are essential for the reconstruction of cardiac imaging.

Fig. (**6**) is an example of reconstructed results obtained by removing the first to sixth components and the reconstructed results obtained by removing the seventh, eighth, ninth, or higher components are also distorted.

Furthermore, the reconstructed image obtained from the removal of the third and fourth components seems to enhance the heart region. The peak of the blue line shifts upward and attenuates the lung region and the valley of the blue line shifts upward, as depicted in Figs. (**6c** and **d**). In addition, the reconstructed image obtained with the removal of the third component is asymmetric imaging.

Therefore, to reconstruct a cardiac EIT image and reduce the influence of the lung caused by expansion, the composition that includes the first, second, third, fifth, sixth, and seventh to twelfth components of the measured data after PCA analysis is adopted to recompose and reconstruct heart imaging, as shown in Fig. (**7**).

Figs. (**7a** and **b**) reveals the use of spheres of FEM with a difference in the radius of the lung to reconstruct an image. It shows a reconstructed image with a fixed heart radius of 0.3 and a lung radius of 0.3 (a) and 0.6 (b). Obviously, the information about the heart region in the reconstructed image has disappeared, as shown in Fig. (**7b**).

Figs. (**7c** and **d**) show the PCA-based reconstructed heart image and the corresponding horizontal center cut of the reconstructed image as depicted in Figs. (**7e** and **f**). The heart region can be reconstructed and separated by PCA even in the case of lung expansion, as shown in Figs. (**7b** and **d**). After observing the impedance change in the horizontal central cut, the peak in the red line shifts downward, and the slope gradient becomes flatter. This indicates that the heart imaging is suppressed when the lung component increases, as shown in Fig. (**7f**). Moreover, the peak of the blue line is the result of the heart imaging after recombination by the PCA analysis.

As shown in Fig. (**7f**), the raised peak of the blue line indicates that the heart region can be reconstructed by PCA-based recombination even when the lung is expanded. The separation effect shown in Fig. (**7e**) is the same as that of Fig. (**7f**).

The PCA-based reconstruction method improves the inhibition of cardiac imaging caused by lung expansion.

#### 3.3.3 EMD-Based Reconstruction

A total of 208 measured data points using the EMD-based method were analyzed and decomposed into six IMFs. One of the six IMFs was removed and the rest were recomposed to reconstruct the image to identify the characteristics of the IMFs.

The reconstructed images and the corresponding horizontal center cut are shown in Figs. (**8** and **9**). In Figs. (**8b, d** and **f**), the reconstructed images with one of the removed IMFs were completely distorted, while the heart and lung region was reversed. The removed components were IMF2, IMF4, and IMF6, respectively. This phenomenon indicates that these components are related to reconstruction. Therefore, these components are required to reconstruct the cardiac image.

Figs. (**8a, c** and **e**) show the horizontal center cut of the reconstructed image obtained by removing the components of IMF1, IMF3, and IMF5 respectively. The resulting impedance change shows the enhanced impedance performance in the heart region. However, there is a varying attenuation in the lung region. All the blue line peaks shifted upward, indicating that the impedance was enhanced in the heart region. Meanwhile, all the blue line valleys shifted upward, which indicates that varying degrees of attenuation were observed in the impedance change in the lung region.

Based on the observation of reconstructed images, it can be deduced that IMF3 and IMF5 are essential for heart image reconstruction. This is because the results indicate that the reconstructed image will be distorted as the IMF3 and IMF5 are removed, as shown in Figs. (**9c** and **e**).

Therefore, IMF2, IMF4, and IMF6, as well as IMF3 and IMF5 are all required to reconstruct the heart image without distortion. To reconstruct the cardiac EIT image and reduce the influence on the lung caused by expansion, the composition (except IMF1) of all the IMFs of the measured data after EMD analysis needs to be adopted to recompose for heart imaging, as shown in Fig. (**10**).

Figs. (**10a** and **b**) demonstrate the use of FEM spheres with a difference in the radius of the lung to reconstruct the image. It shows a reconstructed image of a fixed heart radius of 0.3 and a lung radius of 0.3 (a). It shows a reconstructed image of a heart radius of 0.3 and a lung radius increased to 0.6 (b).

**Fig. (10):**
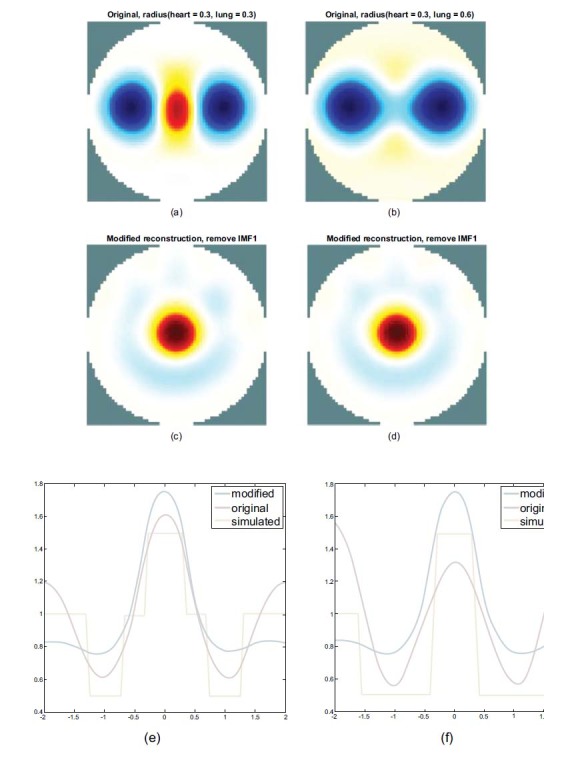
The reconstructed images (**a**, **b**), the EMD-based reconstructed heart images (**c**, **d**), and the comparisons of the corresponding horizontal central cut (**e**, **f**). The radius of the sphere model in the heart is fixed at 0.3, and it is 0.3 (**a**) and 0.6 (**b**) in the lung. EMD: empirical mode decomposition; IMF: intrinsic mode functions.

The information on the heart region in the reconstructed image disappeared, as shown in Figure 10(b).

Figs. (**10c** and **d**) show the EMD-based reconstructed heart image and the corresponding horizontal center cut of reconstructing image as shown in Figs. (**10e** and **f**). The heart region can be reconstructed and separated by EMD even in the case of lung expansion.

It can be observed that with impedance change in the horizontal center cut, the peak of the red line shifts downward and the slope gradient becomes gentle, indicating that the heart imaging is suppressed when the lung radius is increased, as shown in Fig. (**10f**). The peak of the blue line is the result of heart imaging after recombination by EMD analysis.

As shown in Fig. (**10f**), the rising peak of the blue line indicates that the heart region can be reconstructed using the EMD-based recombination even when the lung is expanded. The separation effect shown in Fig. (**10e**) is the same as in Fig. (**10f**).

The EMD-based reconstruction method also improves the inhibition problem of cardiac imaging caused by lung expansion.

### 3.4 Error Estimation

For each heart imaging, the PE, SD, and RNG errors are listed in Table **1**. The phantom experiment assumes the heart and lung radii of 0.3 and 0.6 (unit: arbitrary), respectively. The originally reconstructed image that only shows a part of the lung imaging results in the highest errors of SD and RNG. The PE indicates that both PCA-based and EMD-based heart imaging were reconstructed at the expected position, implying the same PE. Both PCA- and EMD-based methods generate lower SD and RNG errors than the originally constructed image. These errors reveal that the heart region can be depicted without shape deformation and less lung (inverted) information. Moreover, the PCA-based method gives higher RNG errors than the EMD-based one, which reflects a sharper peak of the horizontal center cut,hence, a smaller transition zone. The sharper curve is closer to the curve of the simulated model. This is similar to the expected simulated reconstructed result as shown in Fig. (**7f**) and Fig. (**10f**).

### 3.5 Human Dataset Test

Both PCA and EMD-based methods successfully achieve the reconstruction of heart imaging without distortion after separating the cardiac-related components from the measured data.

Furthermore, we observed an insignificant difference in the enhanced amplitude in the heart region compared to the blue line peaks in Fig. (**7e** and **10e**). The level of the slope gradient is different. In contrast, the slope increases more in the PCA-based method than in the EMD-based method after reconstruction, making it get sharper peaks via the PCA-based method.

In other words, the PCA-based method can make the heart imaging converge at an ideal position that is closer to the simulated model profile.

The results indicate better separation performance in the PCA-based method than in the EMD-based method because of the better accuracy in cardiac imaging.

We applied the PCA-based and EMD-based methods to the human dataset for reconstructing heart images, leading to consistent results as shown in Fig. (**11a**) shows the use of the EIT measured dataset obtained from the Ottawa University and an adult human thorax model to reconstruct image sequence during breathing. Heart imaging is suppressed during breathing because of the effect of lung expansion.

We attempted to reconstruct the heart image based on the rules adopted above-mentioned for recomposing the measured data. As shown in Fig. (**11b**), a part of the heart can be separated from the original measured data using the PCA-based method, and the red region can be reconstructed. Fig. (**11c**) shows that the separated and reconstructed results by the EMD-based method cannot generate the ideal heart image; however, it enhances the heart with distortion.

The section uses two methods to analyze the electrode voltage signal. The heart-related components are retained according to the characteristics of each component, and the lung-related component is removed. Finally, the heart-related signals are recomposed into the modified measured data for reconstruction.

### 3.6 Lung Separation

The impedance changes in the lung are much higher than those in the heart region. It is easier to separate the lung signal from the EIT measured data because the lung signal level is higher than the heart signal level.

In this section, we increased the weighting of the lung-related components according to the component features derived from previous analysis, and the reconstruction of the lung image was performed after the enhancement.

The key component for the EMD-based reconstruction is IMF1, which displays a high correlation to the lung component. However, PC4 is essential to the PCA-based method of reconstruction of the lung image.

### 3.7 EMD-Based Reconstruction

Fig. (**12**) shows an example of the EMD method, which is used to reconstruct the image using different proportions of the lung-related component, IMF1. It shows 0.99 times the IMF1 as shown in Fig. (**12a**), 0 times the IMF1 (b), and 1.01 times of the IMF1 (c).

A part of the heart will appear in the reconstructed image when the composition of IMF1 is reduced (0.99 times) as shown in Fig. (**12a**), and the lung part will begin to be suppressed until only the heart imaging remains (0 times) as shown in Fig. (**12b**).

When the composition of IMF1 increases the weighting by more than 1 times the IMF1, the lung region can be depicted clearly, and the heart region is inhibited, as shown in Fig. (**12c**).

While observing the impedance change in the horizontal center cut, when the composition of IMF1 is reduced from 0.99 times to 0 times of the IMF1, the blue line peak shifts upward, indicating that the heart part is enhanced. The valley of the blue line also shifts upward, revealing that the lung part begins to be suppressed as shown in Figs. (**12a** and **b**). When increasing the weighting to more than 1 times the IMF1, the blue line valley approaches the position of the red line (original in Figure (**12c**)). The lung part can be imaged without distortion, and the blue line peak shifts downward, indicating that the heart part begins to be suppressed as shown in Fig. (**12c**).

Therefore, according to the results, it is possible to reconstruct the undistorted lung image with suppression in the heart by increasing the weighting of the IMF1.

### 3.8 PCA-Based Reconstruction

The same principle is used to adjust the weighting after PCA-based analysis, which uses different proportions of the lung-related component to recompose and further reconstruct the lung image.

Figs. (**13** and **14**) show the results of the use of FEM spheres with different radii of the lung to reconstruct a lung image using the PCA-based method and the EMD-based method. Fig. (**13a**) demonstrates a reconstructed image and a corresponding horizontal center cut of a heart radius of 0.3 and a lung radius of 0.6, using the PCA-based method with the addition of 1% of PC4 and (b) by the EMD-based method with the addition of 1% of IMF1.

Fig. (**14**) shows the reconstructed image of a heart radius of 0.3 and a lung radius decreased to 0.3 using (a) the PCA-based method with the addition of 1% of PC4 and (b) the EMD-based method with the addition of 1% of IMF1.

Figs. (**13a** and **b**) reveal that the reconstruction of a lung image, using the PCA- or the EMD-based method in the state of lung expansion demonstrates an insignificant difference. By observing the horizontal center cut, it can be seen that the blue line peaks shift downward in both the EMD- and PCA-based methods; however, it is slightly lower in the EMD-based method. The valley of the blue line approaches the position of the red line (original), indicating that both the PCA- and EMD-based methods are capable of extracting the lung part.

Figs. (**14a** and **b**) display lung imaging containing some of the heart components while the reconstruction result of a PCA-based method includes more heart components.

Based on the observation of the horizontal central cut, we can conclude that the blue line trends move downward in both methods. However, the decline is more in the EMD-based method than in PCA-based one. This indicates that the PCA-based method will easily be interfered by the heart signal while the lung contracts. Therefore, the EMD-based reconstruction method is more independent of the heart signal and can reconstruct better lung imaging when the lung contracts see Fig. (**14**).

### 3.9 Human Dataset Test

Both the PCA-based and the EMD-based methods of separating lung-related components from the measured data can be successfully achieved to reconstruct the lung image.

Furthermore, we observed that the peak or valley of the blue line shifts downward when the radius of the heart is the same as the lung, indicating that the heart imaging is suppressed and the lung imaging is enhanced, as shown in Fig. (**14**). The separating performance of the EMD is better than that of the PCA because less heart component is included in the reconstructed lung image from the EMD-based method.

We applied the EMD- and PCA-based methods to the human dataset for reconstructing the lung image, leading to consistent results as shown in Fig. (**15**).

Fig. (**15a**) shows the use of the EIT measured dataset obtained from Ottawa University and an adult human thorax model to reconstruct the image sequence during breathing.

Not only does the impedance change when ventilation occurs during breathing, but the cardiac-related impedance also changes.

We attempt to reconstruct the lung image based on the weighting adjustment principle for recomposing the measured data.

As shown in Fig. (**15c**), the lung part can be separated from the original measured data by the EMD-based method to reconstruct the lung image with a slight heart part.

Fig. (**15b**) shows that the separated and the reconstructed results by the PCA-based method still contain more heart-part components.

## 4 DISCUSSION

The goal of this study was to develop a novel method that could separate cardiac- and ventilation-related signals from mixed EIT signals (raw measured voltages) based on EIT simulation and to individually reconstruct the corresponding image without prior knowledge. The major design focused on working on the raw measured data directly instead of reconstructed images sequences. In other words, the proposed method did not need to modify existing EIT reconstruction algorithms such as the GREIT or the Newton-Raphson approach. The separation process works as a preprocessor that keeps the PCA template functions for heart imaging and the IMF of EMD templates for lung imaging and then combines the existing reconstruction algorithm to rapidly obtain individual parts.

Consequently, we performed the novel operation that requires only one set of measured voltages without prior knowledge. We then use both PCA- and EMD-based methods to obtain individual parts before reconstructing the image. The novel operation can be done at a modest computational cost making real-time processing and simultaneous monitoring of perfusion and ventilation possible and feasible.

Individual image extraction of the thorax by the present methods is not yet a feasible clinical solution in real-time mostly because of the difficulty in imaging cardiac-related impedance changes because of the much smaller amplitude and higher conductivity of the cardiac-related component. The image processing in the EIT image sequences is not directly in the raw measured voltages; nevertheless, no cardiac component is obvious in the raw EIT images.

Another problem is that there is no gold standard for the evaluation of the dynamic perfusion-related images such as single-photon emission computed tomography (SPECT) or electron beam computerized tomography (EBCT), which is static. Most studies on the separation of a perfusion image in EIT applying real human data have not assessed the correlations between these methods and a known gold standard. Additionally, there are few studies in the FEM simulation field of view to address this issue of separation.

Against this background, we decided to first form the opinion of FEM simulation to establish EIT phantom to explore this issue, and to develop a reliable method to utilize the developed method on real EIT human data for separation.

### 4.1 FEM Phantom and Method Design

First, the FEM phantom and EIT simulation experiment environments were created, and the phantom was made up of a set of thorax constructions, which included the heart located in the middle surrounding the lungs on both sides.

The heart and lungs parts of material property parameters were chosen from the EIDORS’s recommendation, according to which the heart region was 2 and the lungs region was 0.5. We then simulated lung activity and explored the effect of heart imaging during breathing. The radii of the lungs were designed to vary from 0.3 to 0.6, and that of the heart was fixed at 0.3. See Fig. (**2**)

After the first experimental result, when increasing the lungs part of the FEM model with no change in the heart part, the heart region image disappears in the imaging, and there is no appearance of heart imaging see Figs. (**4a** and **4b**). Additionally, there is no obvious heart image shown in the real EIT images sequences also (see Fig. (**11a**)); the impedance changes in the lungs are dominant for imaging and the heart imaging is suppressed during breathing.

Fig. (**16**) shows the profile of images that display the phenomenon of the attenuated impedance change of the heart region because of the gradual expansion of the lung, and the heart region of profile location is descending from blue to purple location. The impedance change in the heart region should stay unchanged if the radius of the heart is constant.

Hence, the following simulation experiment was performed to choose the extreme FEM model: the radii of the lungs varied from 0.3 to 0.6 while the radius of the heart of 0.3 would be the subject of separation. The known parameters of the FEM model serve as the baseline for the comparison see the red line of Fig. (**4**). The ideal profile of heart imaging location should be approximated to the profile of the sphere model as well as the lung parts and vice versa.

Additionally, in the PCA method, choosing the first 12 principal components as the templates function to recompose meaningful composition could sufficiently reconstruct an image without distortion see Fig. (**5**). In the EMD method, all IMFs were chosen as per the templates function.

Another design procedure was to recompose all the selected components and to adjust the weight of the lung-related key component to determine the new composition for the heart- and lung-related individually reconstructed images.

After the EIT simulation experiment and the analysis of the two methods, the component that is related to the lungs was deconstructed. Moreover, the proportions of the respective key components were adjusted to image the lungs and the heart.

### 4.2 Cardiac-Perfusion-Related Imaging

#### 4.2.1 Principal Component Analysis Based

In Fig. (**6**), the first, second, fifth, and sixth are the combinations of the basic EIT signal needed to reconstruct undistorted images. If one of them is missing, the reconstructed result will be distorted. In Figs. (**6c** and **d**), the removal of the third and fourth showed that the lungs region is greatly weakened, and the entire blue line shifts upwards away from the simulated location while the heart part is highlighted. There is a slight shape deformation in the reconstructed image result of the removal of the third component.

Therefore, the third and fourth components seem to be of a high degree compared with that of lung imaging. The fourth component is the main component related to lung imaging. The third component is one of the principal components containing mixed signals just as the properties of the PCA method cannot completely separate into independent components. The fourth component is the key component that mainly affects lung imaging. Consequently, for heart imaging, we chose to remove the dominant component PC4, which is relative to the signal of lung imaging. Only the heart part appeared in the results of the reconstructed image see Figs. (**7c** and **d**). Fig. (**7**) shows that heart imaging can be reconstructed without the influence of the different sizes of the lung.

In Figs. (**7c** and **d**), both results of the reconstructed heart image show no significant difference. In Figs. (**7e** and **f**), before separation, the entire red line shifts downward, indicating the increasing impedance of the lungs and suppressing heart imaging. In Fig. (**7f**), after PCA-based separation, the entire blue line shifts upward, especially at the peak of the blue line. This indicates that the heart part can be restored and reconstructed.

#### 4.2.2 Empirical Mode Decomposition Based

In Fig. (**8**), IMF 2, 4, and 6 are depicted as the combinations of basic EIT signals needed to reconstruct undistorted images. If one of them is removed, the reconstructed image will be distorted. As the result of removing IMF3, although the blue line shifts upward, indicating the enhancement of the heart part and the attenuation of the lungs parts. The profile of the lungs region is uneven see Fig. (**8c**). Additionally, the reconstructed image corresponding to the removal of IMF3 is also distorted (see Fig. (**9c**)). The removal of IMF5 also shows the distorted result (see Fig. (**9e**)).

Therefore, Fig. (**9a**) shows IMF1 to be of a high degree relative to lung imaging, and the component is the key component for lung imaging.

Consequently, for heart imaging, we chose to remove the dominant component IMF1 relative to the signal of lung imaging. Only the heart part appears in the results of the reconstructed image (see as Figs. (**10c** and **d**)).

Similar to the results of the PCA-based separation from Figs. (**10e** and **f**), before separation, the entire red line shifts downward, indicating the increasing impedance of the lungs and suppressing the heart imaging. In Figs. (**10f**), after the EMD-based separation, the entire blue line shifts upward, especially at the peak of the blue line. Therefore, the heart can also be restored and reconstructed, and the lung parts are weakened at the same time.

Both of the two methods can restore and reconstruct the heart imaging successfully by observing the profile. The result blue line in Figs. (**7** and **10**) of the PCA-based separation approximates more to the simulated part yellow line in Figs. (**7** and **10**) than the EMD-based.

Based on further observation of the error in the reconstruction, there are few differences observed in the cardiac imaging results.

Table **1** shows the high SD 0.98 and high RNG 38.77 errors of the original image because of the main impedance change in lung parts in the reconstructed image see Fig. (**4b**). Compared with the heart sphere model and the heart region of the reconstructed image for the result of heart imaging, it is distorted like shape deformation.

### 4.3 Error Estimation

**Fig. (11):**
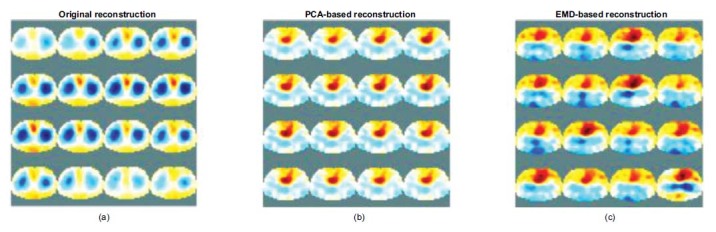
Sequences of the reconstructed images of the lung EIT. The original reconstructed images (**a**) the PCA-based reconstructed heart images (**b**) and the EMD-based reconstructed heart images (**c**). PCA: principal component analysis; EMD: empirical mode decomposition.

The low PE 0.04 and low SD 0.03 errors of the PCA- and EMD-based methods represent the position of the reconstructed heart image. This is expected to be correctly located without shape deformation. The fact that the RNG 1.45 of the PCA-based reconstructions is higher than the RNG 0.77 of the EMD-based reconstructions demonstrates the relative sharpness of the peak of the profile. Therefore, the imaging can rapidly converge at the desired position, and the profile of the reconstructed heart image (blue line) approximates closer to the simulated one (yellow line) (see Figs. (**7f**** and ****10f**)). The slight difference in the slope of the peak in the profile is related for determining whether the heart imaging can converge quickly. The differences in the convergence of the performance reflected in the sequence of the reconstructed images of the human lung data (Figs. **11b**) displays the sequence of the reconstructed heart images using the PCA-based method that is visible as well as the pulmonary-like vascular-related perfusion imaging (blue region). Figs. (**11c**) shows the sequence of the distorted reconstructed heart images by the EMD-based method. The heart region of the heart part seems to spread in the reconstructed images.

Therefore, for the heart-related imaging, using the PCA-based pre-processing yields better results than the EMD-based method. Compared with the findings of separation methods reported in previous studies, the results provide a quick and direct separated and reconstructed method for heart imaging.

### 4.4 Lung-related Imaging

Separating the lung-related signal for lung imaging is easier than the heart part because of the lower level of the conductivity changes of the lung.

The separation strategy adopted the idea that adds 1% of the key component to recompose a new combination for lung imaging. The adjusting component in the PCA-based method is PC4 and IMF1 in the EMD-based method.

Fig. (**12**) demonstrates the reconstructed results, using different proportions of the IMF1. When using 0 times of the IMF1, the heart image can be restored and reconstructed because there is no major lung component used. When it is less by 1 time of the IMF1, the reconstructed image begins to appear at the heart part and slightly suppresses the lung parts. When the IMF1 increases by more than 1.01 times of the IMF1, the reconstructed image shows that only the lungs parts of the corresponding profile approach the original curve.

Fig. (**13**) compares the results of the two methods of the reconstruction of lungs images when the radius of lungs is greater than that of the heart. There is no significant difference in the reconstructed image.

However, when the radii of lungs and heart are not very different, the heart component in the reconstructed image begins to appear see Fig. (**14**), especially in the result of the PCA-based processing. The heart component also seems to interfere with lung imaging when decreasing the lung part. The interference of the heart component is indeed reflected in the reconstructed images of the human EIT data see Figs. (**15b**) displays the heart parts in the reconstructed lung images sequence, using the PCA-based method. In Fig. (**15c**), nevertheless, almost all the lung parts can be reconstructed and depicted with less interference of the heart component.

As mentioned in the introduction, V/Q matching is the key role in lung physiology and the lung EIT is a promising tool for real-time monitoring at bedside. Many situations wherein lung EIT will be helpful and feasible to reveal the V/Q matching mapping in the region of lungs, e.g., ARDS causing the V/Q mismatching in the region of the lung during PEEP titration.

However, we successfully separated and reconstructed individual images in EIT simulation and implemented it in healthy EIT data at first. Separation under different physiological conditions is still on the dark side and needs to be thoroughly analyzed and investigated further.

## CONCLUSION

In the raw EIT reconstructed images, the respiratory- and cardiac-related conductivity changes were measured simultaneously. However, it is difficult to extract cardiac-perfusion-related conductivity changes from mixed EIT images.

This study proposed methods that extract different components from the raw measured data instead of the raw EIT images and further reconstruct spatial conductivity distributions of perfusion- and ventilation-related imaging.

To the best of the authors’ knowledge, the proposed method is the first from the EIT simulation field that explores the impedance changes in the thorax and attempts to reconstruct the EIT images from the individual components.

This novel method is capable of effectively recovering the cardiac-perfusion-related images from the set of images that are inherently lung-ventilator-related.

When both PCA- and EMD-based methods are used as pre-processors, they can generate a modified combination for reconstructing the images. The PCA-based pre-processing is sensitive to impedance changes in the heart while performing lung imaging, especially when the lung is contracting. Meanwhile, the EMD-based pre-processing is sensitive to impedance changes in the lung while carrying out heart imaging. This makes the reconstructed result distorted in the real lung EIT data.

Nevertheless, the validation of perfusion images is often challenging and limited. Therefore, it should be noted that, because the blood pressure signal was gathered and compared with separated perfusion images, this study only considered feasible separation cases. Furthermore, solely the phantom simulation in EIT was used to validate the effectiveness of image separation.

The method proposed in this study combines PCA-based methods for heart imaging and EMD-based methods for lung imaging and inputs one set of the EIT measured data to easily obtain two individual parts. This paves the way for clinical applications of lung EIT that can focus on V/Q matching based on the proposed novel method. In this study, the EIT data were obtained from healthy adults who could breathe normally. Future studies will focus on non-healthy patients having different physiological conditions (such as adult respiratory distress syndrome or lung emboli) related to V/Q matching.

## Figures and Tables

**Fig. (1):**
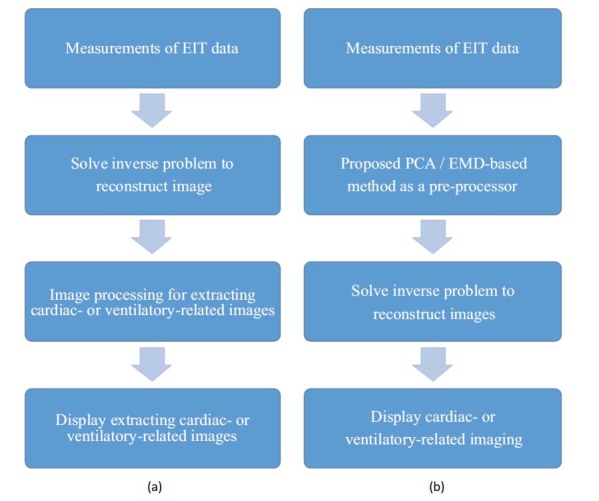
Comparison between the existing method and the proposed method. (**a**) General reconstruction method with separation procedure as post-processing. (**b**) The proposed method with separation procedure as pre-processing. EIT: Electrical impedance tomography; PCA: principal component analysis; EMD: empirical mode decomposition.

**Fig. (2):**
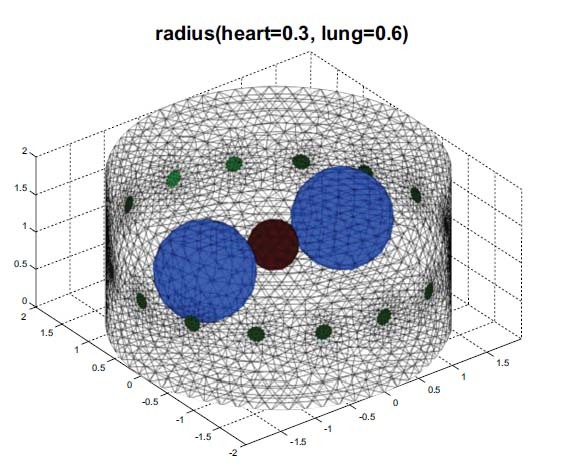
EIDORS spheres Finite Element Method FEM phantom in 3D.

**Fig. (3):**
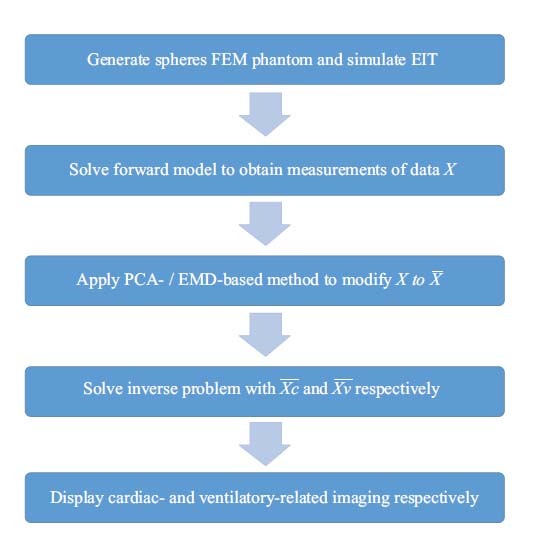
The flowchart for generating phantom data and separating desired voltages for EIT reconstruction. EIT: Electrical impedance tomography; PCA: principal component analysis; EMD: empirical mode decomposition; FEM: Finite Element Method.

**Fig. (4):**
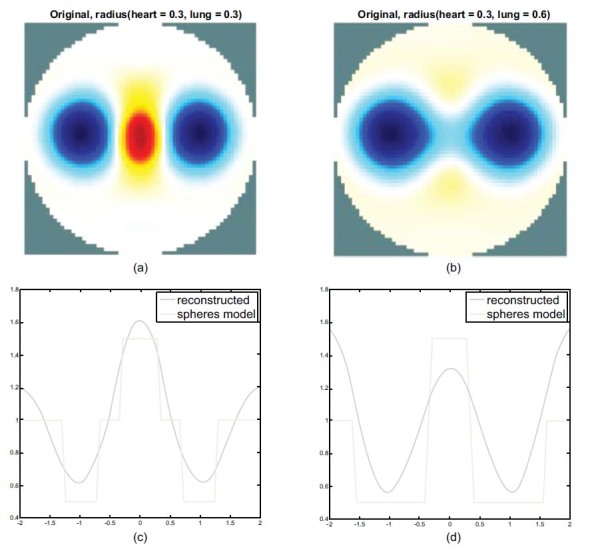
Reconstructed images and the corresponding horizontal central cut. The radius of the sphere model in the heart is fixed at 0.3 and that of the lung is fixed at 0.3 (**a**) and 0.6 (**b**). The corresponding profile of the images and the sphere models are shown in (**c**) and (**d**).

**Fig. (5):**
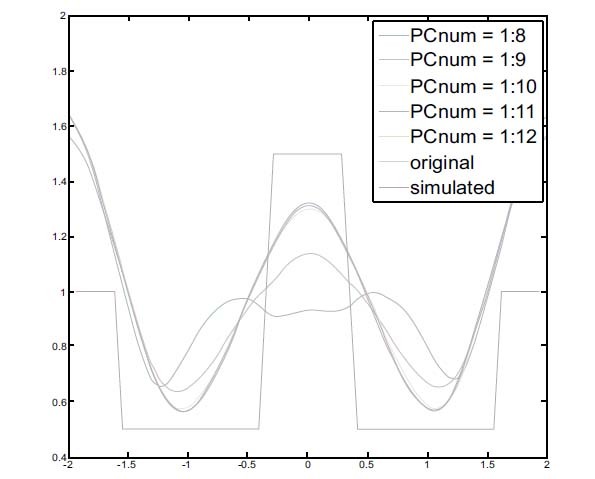
The horizontal central cut of PCA-based reconstructed images with different ratios of PCnums. PCA: principal component analysis.

**Fig. (6):**
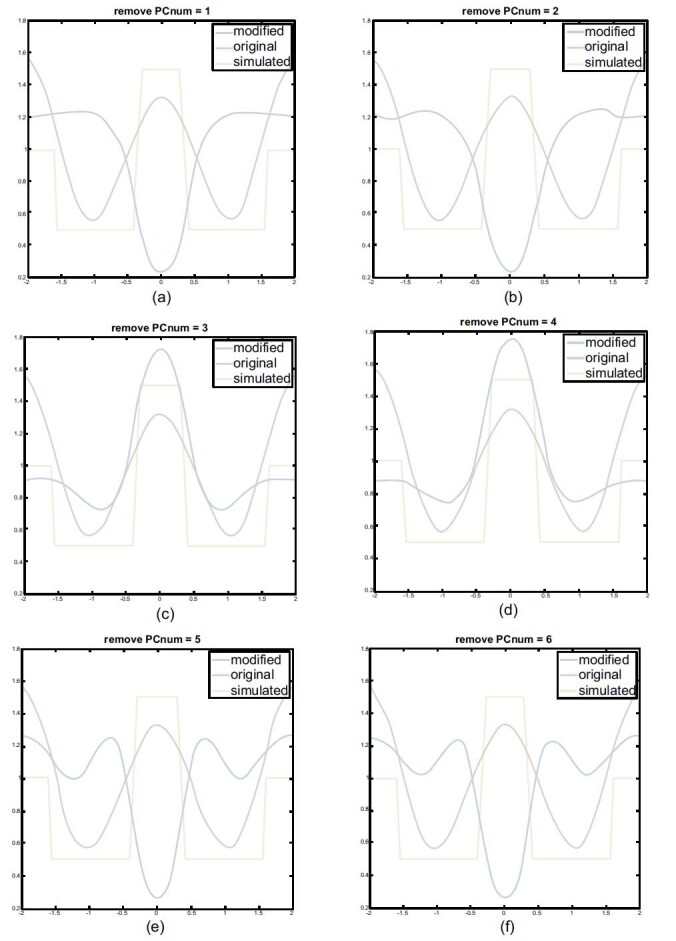
The horizontal central cut of PCA-based reconstructed images using different combinations. PCA: principal component analysis.

**Fig. (7):**
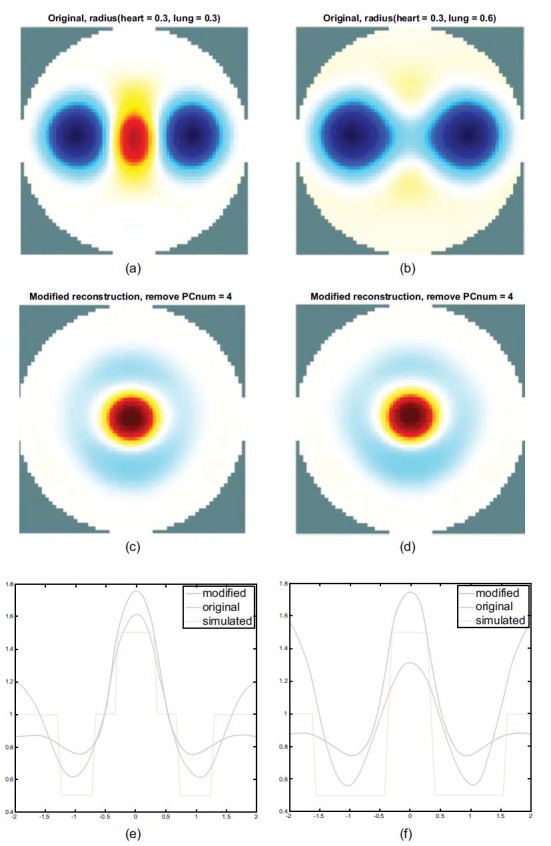
The reconstructed images (**a**, **b**), the PCA-based reconstructed heart images (**c**, **d**), and a comparison of the corresponding horizontal central cut (**e**, **f**). The radius of the sphere model in the heart is fixed at 0.3, and in the lung, it is 0.3 (**a**) and 0.6 (**b**). PCA: principal component analysis.

**Fig. (8):**
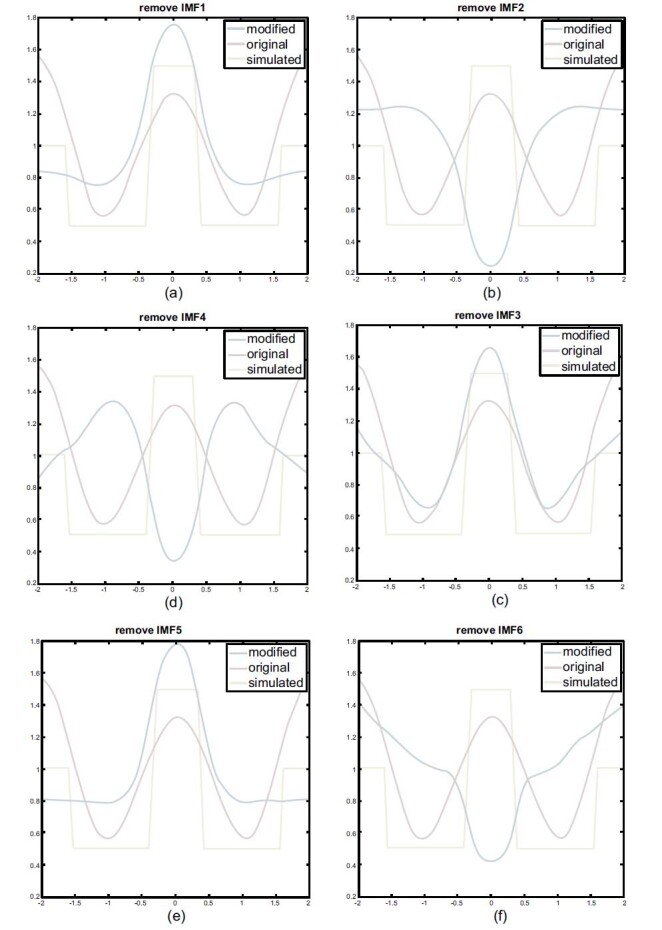
Horizontal central cut of the EMD-based reconstructed images using different combinations. EMD: empirical mode decomposition; IMF: intrinsic mode functions.

**Fig. (9):**
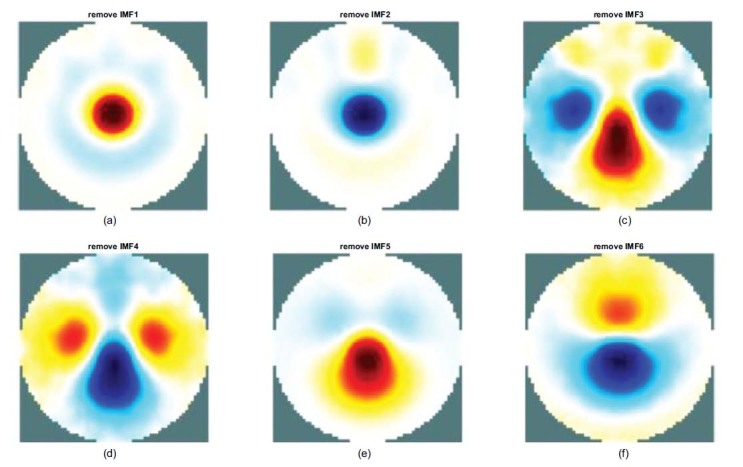
EMD-based reconstructed images using different combinations. EMD: empirical mode decomposition; IMF: intrinsic mode functions.

**Fig. (12):**
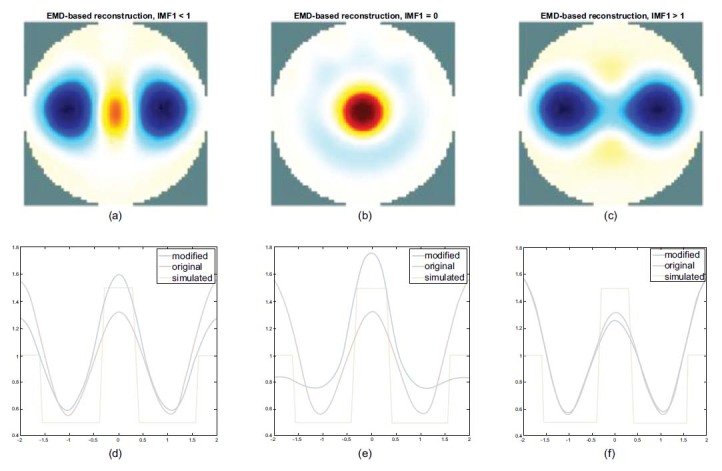
Exemplary EMD-based reconstructed images and the corresponding horizontal central cut using varied proportions of the IMF 1. Multiply the IMF1 by the weighting of 0.99 (**a**), 0 (**b**), and 1.01 (**c**). EMD: empirical mode decomposition; IMF: intrinsic mode functions.

**Fig. (13):**
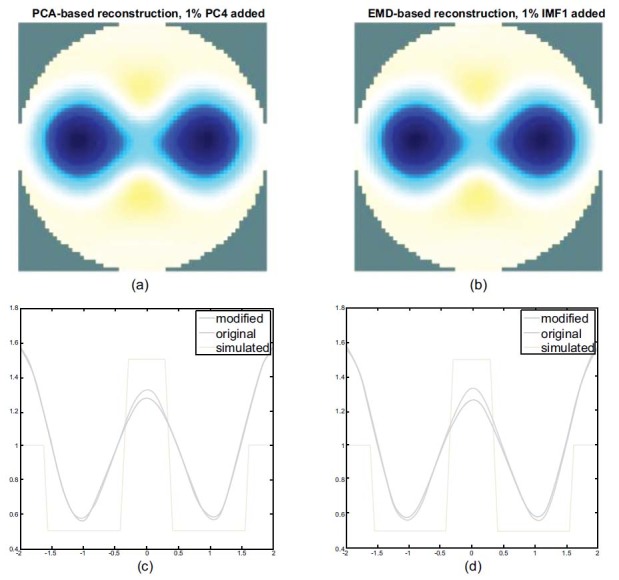
Comparison of the PCA- and the EMD-based reconstructed lung images. The radius of the sphere models in the heart is fixed at 0.3 and is 0.6 in the lung. The PCA-based reconstructed lungs image with 1% of PC4 added (**a**) and the EMD-based reconstructed lungs image with 1% of IMF1 added (**b**). PCA: principal component analysis; EMD: empirical mode decomposition.

**Fig. (14):**
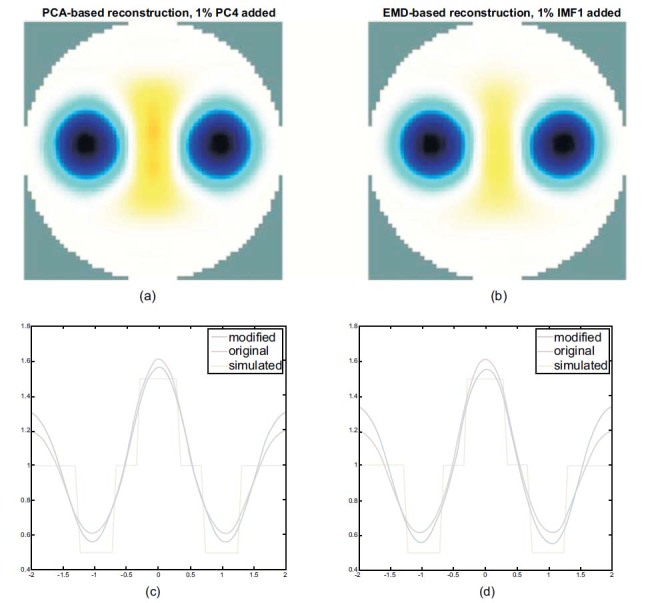
Comparison of the PCA- and EMD-based reconstructed lung images. The radius of the sphere models in the heart is fixed at 0.3, and that is 0.3 in the lung. The PCA-based reconstructed lungs image with 1% of PC4 added (**a**) and the EMD-based reconstructed lungs image with 1% of IMF1 added (**b**). PCA: principal component analysis; EMD: empirical mode decomposition.

**Fig. (15):**
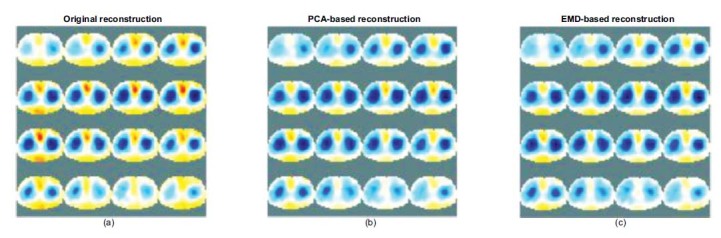
Sequences of reconstructed images of the lung EIT. The original reconstructed images (**a**), the PCA- (**b**), and EMD-based reconstructed lung images (**c**). EIT: Electrical impedance tomography; PCA: principal component analysis; EMD: empirical mode decomposition.

**Fig. (16):**
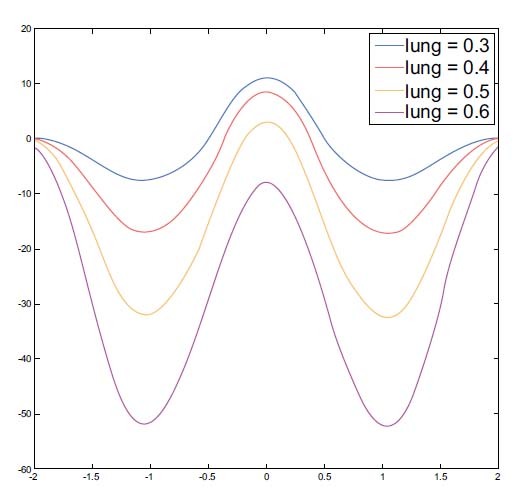
The horizontal center cut of reconstructed images based on varying radii of the lung.

**Table 1:** PE, SD and RNG errors obtained from the phantom measurements with spheres.

**Heart Imaging**	**Original**	**PCA-Based**	**EMD-Based**
PE	0.04	0.04	0.04
SD	0.98	0.03	0.02
RNG	38.77	1.45	0.77

Corresponding images are shown in Figs. (**7** and **10**)

PE: Position error; SD: Shape deformation; RNG: Ringing; PCA: principal component analysis; EMD: empirical mode decomposition; FEM: Finite Element Method.

## Data Availability

All relevant data are acquired from the following source: https://sourceforge.net/p/eidors3d/code/HEAD/tree/trunk/eidors/sample_data/montreal_data_1995.mat. Andy Adler, William R B Lionheart (2006) “Uses and abuses of EIDORS: An extensible software base for EIT”, Physiol. Meas. 27:S25-S42, 2006
